# Supplementary material for: Different Characteristics and Clinical Outcomes between Early-Onset and Late-Onset Asthma: A Prospective Cohort Study
Source: J Clin Med. 2022 Dec 9;11(24):7309. doi: 10.3390/jcm11247309 (PMC9785577; doi:10.3390/jcm11247309)
Supplement: Supplementary file 1 [file jcm-11-07309-s001.zip › jcm-2049492-supplementary.pdf]

Journal of Clinical Medicine

MDPI

Table S1. Characteristics of patients (<65 years old) with early-onset and late-onset asthma.

Variables	EOA (*n* = 15)	LOA (*n* = 28)	*p*-Value
Age, years, mean (SD)	39±11.1	56.0±7.0	<0.0001
Male, *n* (%)	7(46.7%)	13(46.4%)	0.988
Body mass index, kg/m^2	25.4±2.5	25.0±4.6	0.747
Non-smoker, *n* (%)	9(60%)	11(39.3%)	0.194
ACT score, mean (SD)	19.9±5.7	21.3±4.5	0.389
Age at asthma onset, years,	24.5±10.9	52.3±7.0	<0.0001
Duration of asthma, years,	14.5±14.8	3.8±4.5	0.015
Family history of asthma, *n* (%)	8(53.3%)	7(25%)	0.063
Comorbidities			
Gastroesophageal reflux, *n* (%)	10(66.7%)	15(53.6%)	0.407
Allergic rhinitis, *n* (%)	13(86.7%)	16(57.1%)	0.049
Rhinosinusitis with or without polyps, *n* (%)	7(46.7%)	7(25%)	0.148
Aspirin sensitivity, *n* (%)	2(13.3%)	3(10.7%)	0.798
Anxiety or depression, *n* (%)	6(40%)	13(46.4%)	0.686
Obstructive sleep apnea, *n* (%)	2(13.3%)	9(32.1%)	0.178
Allergic status			
IgE level, kU/L, mean (SD)	215±225.8	502.3±887.2	0.228
ECP level, μg/L, mean (SD)	15.6±11.0	17.9±22.1	0.714
Eosinophil count, cells/μL	293.4±204.2	324.2±355	0.794
Atopy, *n* (%)	10(66.7%)	14(50%)	0.217
Fungus sensitization	2(13.3%)	3(10.7%)	0.967
Baseline pulmonary function test			
FVC, % of prediction	83.8±15.4	81.1±13.5	0.562
FEV_1, % of prediction	75.7±21.8	70.1±17.4	0.367
FEV_1/FVC (%)	75.1±14.4	71.2±11.0	0.331
Treatment			
ICS, *n* (%)	1(6/7%)	1(3.6%)	0.646
ICS+LABA, *n* (%)	14(93.3%)	24(85.7%)	0.458
ICS+LABA+LAMA, *n* (%)	1(6.7%)	4(14.3%)	0.458
Montelukast, *n* (%)	7(46.7%)	19(67.9%)	0.176
OCS, *n* (%)	3(20%)	6(21.4%)	0.913
Biologics, *n* (%)	1(6.7%)	1(3.6%)	0.646

Abbreviations: EOA: early-onset asthma (age of onset < 40 year of age); LOA: late-onset asthma; ACT: asthma control test; FVC: forced vital capacity; FEV_1: forced expiratory volume in one second; IgE: immunoglobulin E; ECP: eosinophil cationic protein; Atopy: any positive specific IgE; ICS: inhaled corticosteroids; LABA: long-acting beta 2 agonist; LAMA: long-acting muscarinic antagonist; and OCS: oral corticosteroids.

Table S2. Clinical outcomes of patients (<65 years old) with asthma after 12-month treatment.

Variables	EOA (*n* = 15)	LOA (*n* = 28)	*p*-Value
ACT score, mean (SD)	21.5±6.4	23.5±3.0	0.391
Mean difference from baseline	−1.8±7.0	3.2±4.7	0.118
FVC, % of prediction	64.3±23.2	88.2±16.4	0.10
Mean difference from baseline	−8.0±12.7	8.4±11.9	0.008
FEV_1, % of prediction	49.3±15.6	68.0±21.8	0.007
Mean difference from baseline	−5.3±8.0	10.2±12.3	0.008
FEV_1/FVC (%)	65.7±15.9	70.8±13.1	0.429
Mean difference from baseline	3.4±13.4	−0.1±8.3	0.454
Exacerbations in the past year	5(33.3%)	10(35.7%)	0.876
Systemic corticosteroid bursts	3(20%)	6(21.4%)	0.913
Emergency department visits	2(13.3%)	5(17.9%)	0.702
Hospitalization	3(20%)	2(7.1%)	0.210
Exacerbations during the 12 months	3(20%)	4(14.3%)	0.629
Systemic corticosteroid bursts	3(20%)	4(14.3%)	0.629
Emergency department visits	0(0%)	1(3.6%)	0.459
Hospitalization	0(0%)	0(0%)	NA

Abbreviations: EOA: early-onset asthma (age of onset < 40 year of age); LOA: late-onset asthma; ACT: asthma control test; FVC: forced vital capacity; FEV_1: forced expiratory volume in one second;.

Table S3. Characteristics of patients (≥ 65 years old) with early-onset and late-onset asthma.

Variables	EOA (*n* = 6)	LOA (*n* = 52)	*p*-Value
Age, years, mean (SD)	72.2±9.4	73.9±7.2	0.588
Male, *n* (%)	5(83.3%)	34(65.4%)	0.375
Body mass index, kg/m^2	26.0±4.6	25.3±4.0	0.667
Non-smoker, *n* (%)	2(33.3%)	26(50%)	0.439
ACT score, mean (SD)	21.8±5.7	21.7±3.7	0.951
Age at asthma onset, years,	20.5±10.8	67±10.3	<0.0001
Duration of asthma, years,	51.7±12.9	6.9±8.5	<0.0001
Family history of asthma, *n* (%)	2(33.3%)	13(25%)	0.659
Comorbidities			
Gastroesophageal reflux, *n* (%)	2(33.3%)	22(42.3%)	0.673
Allergic rhinitis, *n* (%)	5(83.3%)	27(51.9%)	0.143
Rhinosinusitis with or without polyps, *n* (%)	3(50%)	9(17.3%)	0.061
Aspirin sensitivity, *n* (%)	1(16.7%)	1(1.9%)	0.061
Anxiety or depression, *n* (%)	2(33.3%)	28(53.8%)	0.341
Obstructive sleep apnea, *n* (%)	1(16.7%)	14(26.9%)	0.587
Allergic status			
IgE level, kU/L, mean (SD)	234.5±327.2	229.4±345.4	0.973
ECP level, µg/L, mean (SD)	9.3±8.4	8.3±10.0	0.817
Eosinophil count, cells/µL	178±134.1	163.8±126.2	0.912
Atopy, *n* (%)	6(100%)	41(78.8%)	0.094

Fungus sensitization	1(16.7%)	14(26.9%)	0.412
Baseline pulmonary function test			
FVC, % of prediction	87.8±14.6	73.7±21.5	0.160
FEV_1, % of prediction	76.6±23.7	66.9±23.4	0.381
FEV_1/FVC (%)	67.3±11.1	71.1±15.8	0.598
Treatment			
ICS, *n* (%)	0(0%)	1(1.9%)	0.732
ICS+LABA, *n* (%)	4(66.7%)	44(84.6%)	0.270
ICS+LABA+LAMA, *n* (%)	1(16.7%)	10(19.2%)	0.879
Montelukast, *n* (%)	2(33.3%)	14(26.9%)	0.739
OCS, *n* (%)	0(0%)	3(5.8%)	0.546
Biologics, *n* (%)	0(0%)	1(1.9%)	0.732

Abbreviations: EOA: early-onset asthma (age of onset < 40 year of age); LOA: late-onset asthma; ACT: asthma control test; FVC: forced vital capacity; FEV_1: forced expiratory volume in one second; IgE: immunoglobulin E; ECP: eosinophil cationic protein; Atopy: any positive specific IgE; ICS: inhaled corticosteroids; LABA: long-acting beta 2 agonist; LAMA: long-acting muscarinic antagonist; and OCS: oral corticosteroids.

Table S4. Clinical outcomes of patients (≥ 65 years old) with asthma after 12-month treatment.

**Variables**	**EOA (*n* = 6)**	**LOA (*n* = 52)**	***p*-Value**
ACT score, mean (SD)	24.6±0.6	23.7±1.5	0.218
Mean difference from baseline	0.8±2.4	1.3±2.7	0.727
FVC, % of prediction	87.4±20.3	79.4±25.8	0.524
Mean difference from baseline	−0.4±10.9	4.0±14.9	0.538
FEV_1, % of prediction	79.4±26	67.3±11.1	0.426
Mean difference from baseline	1.8±7.9	3.9±13.3	0.739
FEV_1/FVC (%)	69.3±6.5	66.8±15.4	0.726
Mean difference from baseline	2.0±5.8	0.6±5.2	0.596
Exacerbations in the past year	0(0%)	14(26.9%)	0.145
Systemic corticosteroid bursts	0(0%)	4(7/7%)	0.481
Emergency department visits	0(0%)	6(11.5%)	0.380
Hospitalization	0(0%)	7(13.5%)	0.338
Exacerbations during the 12 months	1(16.7%)	5(9.6%)	0.591
Systemic corticosteroid bursts	1(16.7%)	5(9.6%)	0.551
Emergency department visits	0(0%)	0(0%)	NA
Hospitalization	0(0%)	0(0%)	NA

Abbreviations: EOA: early-onset asthma (age of onset < 40 year of age); LOA: late-onset asthma; ACT: asthma control test; FVC: forced vital capacity; FEV_1: forced expiratory volume in one second.
